# Chlorogenic acid alleviates renal fibrosis by reducing lipid accumulation in diabetic kidney disease through suppressing the Notch1 and Stat3 signaling pathway

**DOI:** 10.1080/0886022X.2024.2371988

**Published:** 2024-07-02

**Authors:** Xiao-ying Yang, Die Jiang, Yuan-zhu Wang, Mei-yan Duan, Ye-wei Huang, Xuan-jun Wang, Ze-min Xiang, Jun Sheng, Qiang-qiang Zhu

**Affiliations:** a Key Laboratory of Pu-er Tea Science, Ministry of Education, Yunnan Agricultural University, Kunming, China; b College of Science, Yunnan Agricultural University, Kunming, China; c College of Food Science and Technology, Yunnan Agricultural University, Kunming, China; d State Key Laboratory for Conservation and Utilization of Bio-Resources in Yunnan, Kunming, China

**Keywords:** Chlorogenic acid, diabetic kidney disease, renal fibrosis, renal lipid accumulation, Notch1, Stat3

## Abstract

**Aims:**

Abnormal renal lipid metabolism causes renal lipid deposition, which leads to the development of renal fibrosis in diabetic kidney disease (DKD). The aim of this study was to investigate the effect and mechanism of chlorogenic acid (CA) on reducing renal lipid accumulation and improving DKD renal fibrosis.

**Methods:**

This study evaluated the effects of CA on renal fibrosis, lipid deposition and lipid metabolism by constructing *in vitro* and *in vivo* models of DKD, and detected the improvement of Notch1 and Stat3 signaling pathways. Molecular docking was used to predict the binding between CA and the extracellular domain NRR1 of Notch1 protein.

**Results:**

*In vitro* studies have shown that CA decreased the expression of Fibronectin, α-smooth muscle actin (α-SMA), p-smad3/smad3, alleviated lipid deposition, promoted the expression of carnitine palmitoyl transferase 1 A (CPT1A), and inhibited the expression of cholesterol regulatory element binding protein 1c (SREBP1c). The expression of Notch1, Cleaved Notch1, Hes1, and p-stat3/stat3 were inhibited. These results suggested that CA might reduce intercellular lipid deposition in human kidney cells (HK2) by inhibiting Notch1 and stat3 signaling pathways, thereby improving fibrosis. Further, *in vivo* studies demonstrated that CA improved renal fibrosis and renal lipid deposition in DKD mice by inhibiting Notch1 and stat3 signaling pathways. Finally, molecular docking experiments showed that the binding energy of CA and NRR1 was −6.6 kcal/mol, which preliminarily predicted the possible action of CA on Notch1 extracellular domain NRR1.

**Conclusion:**

CA reduces renal lipid accumulation and improves DKD renal fibrosis by inhibiting Notch1 and stat3 signaling pathways.

## Introduction

Diabetes is one of the most common chronic diseases in the world, and it is estimated that by 2045, the number of people with diabetes will rise to 12.2% (783.2 million) [1]. Various complications occur with the progression of diabetes, among which diabetic kidney disease (DKD) is one of its major complications, which is characterized by the accumulation of extracellular matrix eventually leading to renal tubulointerstitial fibrosis and glomerulosclerosis [2]. Inflammation and oxidative stress play a key role in DKD, and there have been a lot of studies on anti-inflammatory and antioxidant stress to reduce DKD [3]. In recent years, a large number of studies have shown that triglyceride levels in the kidneys of nephropathy patients and diabetic animal models are elevated, and in human DKD, renal lipid accumulation is associated with renal dysfunction [4, 5]. More and more evidence shows that there is abnormal lipid metabolism and glomerular lipid deposition in DKD [6, 7] and glomerular and renal tubule lipid accumulation contributes to the development of type 2 diabetes-related kidney injury [8]. Therefore, reducing renal lipid accumulation is a strategy to treat DKD.

Chlorogenic acid (CA), also known as 5-caffeoylquinic acid (5-CQA) [9], is a phenolic compound, this compound is not only found in coffee, fruits and vegetables, but also in traditional Chinese medicine preparations [10, 11]. It has been found to play a role in lowering blood sugar [12, 13], lowering blood lipids [14], anti-bacterial [15], anti-oxidant [16], anti-inflammatory [17]. However, there is little about the anti-DKD effect of CA is reported in the study. CA prevents DKD mainly by inhibiting oxidative stress and inflammation [18], At present, there is no study on the regulation of renal lipid accumulation by CA to alleviate DKD.

Notch signaling is a highly conserved signaling pathway that regulates cell proliferation, differentiation, apoptosis [19], and inflammation [20]. After Notch ligands bind to receptors, Notch intracellular domain (NICD) is cleaved by protease, and the cleaved Notch intracellular domain (NICD) acts as a Notch downstream signaling factor to induce the transcription of the target gene Hes1 in the nucleus, thereby inducing the occurrence of related diseases [21]. Previous studies have reported that inhibition of Notch signaling pathway in fatty liver regulates lipid metabolism to improve fatty liver [22], and Notch1 loss reduces lipid accumulation in liver by inducing fatty acid oxidation [23]. In nephropathy, Notch’s role in promoting renal fibrosis is related to inhibition of fatty acid metabolism in renal tubular epithelial cells [24]. However, there have been no studies on small molecule compounds that inhibit Notch signaling pathway to regulate lipid metabolism and improve DKD. In addition, JAK/STAT is also an important cross-signaling cascade in the pathogenesis of DKD [25]. There is evidence that inhibiting stat3 activation in STZ-induced DKD can prevent the progression of fibrosis [26] and inhibited Stat3 signal can promote fatty acid oxidation and thus improve lipid accumulation in the liver.[27]. Moreover, cross-talk between Notch-Hes and stat3 signaling has also been reported [28]. Thus, we hypothesized that CA may attenuate DKD in diabetic mice by targeting Notch1 and stat3 signaling pathway.

In this study, we investigated the effects of small molecule compound CA on fibrosis and renal lipid accumulation in a high-glucose and high-lipid induced renal tubular epithelial cell (HK2) model and HFD/STZ-induced mouse model of type 2 diabetes, and revealed the related mechanisms. The results suggest that CA reduces fibrosis and lipid accumulation in part by inhibiting Notch1 and stat3 signaling pathways, thereby improving the progression of DKD.

## Materials and methods

### Cells, antibodies and reagents

Human proximal tubular epithelial cells (HK-2) were stored in Key Laboratory of Pu -er Tea Science of Ministry of Education. D-glucose and Palmitic acid (PA) were purchased from Sigma-Aldrich (USA). Chlorogenic acid (CA) was purchased from Chengdu Prifa Biotechnology Co., LTD(China). Streptozotocin (STZ), Metformin (Met), MTT (3-(4-5-Dimethylthiazol-2-yl-2,5-diphenyltetrazolium bromide) were purchased from Solarbio (China). Antibodies against Fibronectin, α-SMA, p-smad3, smad3, β-tubulin and β-actin were purchased from ABclonal (China). CPT1A, SREBP1c were purchased from Proteintech (China). Notch1, Hes1, p-stat3, stat3 antibodies were purchased from Cell signaling technology (USA). Cleaved Notch1 antibodies were purchased from Thermo fisher.

### Cell viability assay

The MTT assay was utilized to evaluate CA on cell viability. The HK2 in the logarithmic growth period were collected and seeded into a 96-well plate at 1 × 10^4^ cells/well. HK-2 cells were seeded into in 96-well plates and incubated overnight to allow the cells to adhere. Subsequently, Cells were exposed to same to the condition used for cell culture study, cells were divided into eight groups as follows: (1)normal glucose(NG, 5.6 mM), (2)High glucose (HG,30mM)+Palmitic acid (PA, 0.2 mM), (3)HG + PA + CA(20 μM),(4)HG + PA + CA(40 μM),(5)HG + PA + CA(80 μM),(6)HG + PA + CA(120 μM), (7) HG + PA + CA(160 μM),(8)HG + PA + CA(200 μM). Each group was treated for 24 h, and then, 20 μL of MTT was added and incubated for another 4 h. The supernatant was discarded, and formazan products were dissolved in DMSO. The absorbance at 492 nm was measured using a microplate reader.

### Cell culture and treatment

We cultured and built cell models according to previous methods [29, 30]. HK2 were cultured in DMEM-F12 medium containing 10% FBS, 1% penicillin and 1% streptomycin at 37 °C in a 5% CO2 incubator. Cells were trypsinized and passaged when they grew to 70–80% confluency, and cells were inoculated with 6-well plates and 12-well plates, respectively. Subsequently, HK2 cells were grouped after adhesion. cells were divided into five groups as follows: (1) normal glucose (NG, 5.6 mM), (2) High glucose (HG, 30mM)+Palmitic acid (PA, 0.2 mM), (3) HG + PA + CA (20 μM), (4) HG + PA + CA (40 μM), (5) HG + PA + CA (80 μM). Each group was treated for 24 h. The 6-well plates were used for immunoblotting, and the 12-well plates (Cell creep) were used for Nile red and oil red O staining.

### Animal experimental design

All animal procedures were performed in accordance with the Guidelines for Care and Use of Laboratory Animals of Yunnan Agricultural University and were approved by the Animal Ethics Committee of Yunnan Agricultural University (YNAU2019LLWYH003-3). A total of 8-week-old C57BL/6 mice (∼25 g B.W) were purchased from Henan Skobes Biotechnology Co., LTD (SCBS, China), then they were divided into four groups. Each group had six mice. The first group was fed with a normal diet for 12 weeks until the end of the experiment, The second group, the third group and fourth group were fed with a high fat diet (HFD, 60% fat supply high fat purified feed, Jiangsu Synergetics Biological Engineering Co., LTD, China). After 4 weeks of HFD feeding, mice of group 2, group 3 and group 4 were single injected intraperitoneally with Streptozotocin (STZ) for once (100 mg/kg B.W, dissolved in cold citrate buffer, 0.05 M, pH 4.5). The blood glucose level of each mouse was measured using tail-vein blood samples utilizing a glucose meter (Sannuo, Changsha, China). Mice having a blood glucose level above 16.7 mmol/L after 72 h of STZ injection were considered as diabetic mice and maintained for up to 8 weeks to develop DKD. Moreover, the third group and fourth group mice were administered CA (dissolved in PEG400: drinking water =3: 2, 50 mg/kg) and Met (dissolved in the drinking water, 50 mg/kg) respectively every day up to 8 weeks. blood glucose level and body weight were measured every two weeks during administration. After 8 weeks of treatment, mice were placed in metabolic cages to collect 24 h urine, and then all mice were sacrificed, blood and kidneys were collected.

### Determination of urinary protein, serum total cholesterol (TG) and triglyceride (TC)

Urine was collected from metabolic cages, then centrifuged at 3,500 rpm for 15 min at 4 °C, and urinary protein were measured with urine protein test kit (Nanjing Jiancheng Bioengineering Research Institute, China). The collected blood was left to stand for more than 30 min and centrifuged at 3,500 rpm for 15 min at 4 °C. The TG and TC of the blood serum were measured by Total Cholesterol detecting kit and triglyceride detecting kit (Nanjing Jiancheng Bioengineering Research Institute, China).

### Oil red O and Nile red staining

Oil red O staining and Nile red (Yeasen, China) staining were performed according to the Manufacturer’s specification on 8 μm-thick sections of frozen kidney tissue or cell creep. The oil red O kidney tissue samples were fixed in 4% paraformaldehyde, submerged in oil red O stain solution (1320-06-5, Sigma) for 10 min and counterstained in hematoxylin stain solution (Sigma, USA) for 1 min and then washing sections. The oil red O samples (cell creep) were stained according to the cell specific oil red O staining kit (G1262, solarbio). The Nile red samples (cell creep) were rinsed with PBS and stained with Nile red working solution (10 μg/ml) for 10 min at room temperature and cell nuclei were counterstained DAPI. The stained samples were imaged with a general microscope (CX21, Leica) or a fluorescence microscope (DM2000, Leica). Oil red O slides were scanned at 400× magnification. Nile red slides were scanned at 200× magnification.

### H&E and Masson staining

H&E and Masson staining were performed on frozen sections of 8 μm thickness according to manufacturer’s instructions. The kidney tissue sections were randomly selected and stained with hematoxylin (Sigma, USA) and eosin (Solarbio) to evaluate histopathological damage (renal tubular dilatation, The basal membrane of the glomerulus thickens), and also stained with Masson’s trichrome kit (Solarbio) for collagen fibers. Images were captured using a Leica microscope and Slides were scanned at 400× magnification.

### Immunohistochemistry

The frozen section with thickness of 8 μM was selected for the immunohistochemical test of Notch1, Hes1 protein. The avidin–biotin complex kit (Vector Laboratories, Burlingame, USA) and 3,3’-diaminobenzidine kit (Tianjin, Beijing, China) were used for staining according to the manufacturer’s instructions. Slides were scanned at 400× magnification.

### Western blot analysis

HK2 cells and renal tissue protein were extracted with RIPA lysate (Solarbio, China), the total protein concentration was determined according to BCA kit (Solarbio, China), and the 5× reduction carrier solution was mixed with the sample and boiled in gold water at 95 °C for 10 min. Then, the sample protein was isolated by SDS-PAGE and transferred to PVDF membrane (Sigma), and sealed with 5% skim milk powder for 1 h. After the closure of the sealing solution, the relevant primary antibody was incubated for 16h. the primary antibody was recovered, and the corresponding secondary antibody (mouse or rabbit antibody) was incubated for another 1h, and finally the exposure was performed.

### Molecular docking

Molecular docking was performed using AutoDock 4.2 as previously described [31]. The crystal structure of NRR1 (Extracellular domain of transmembrane receptor protein Notch1) was acquired from Protein Data Bank. The CA minimization was performed with MOE before analysis. Discovery Studio 4.5 Visualizer was used to analyze the interaction between CA and NRR1.

### Statistical analysis

Data were expressed as mean ± SEM. Statistical analysis was performed by one-way ANOVA. Statistical significance was defined as *p* < 0.05.

## Results

### CA inhibits hyperglycemic and hyperlipid-induced fibrosis in HK2 cells

**Figure 1.**
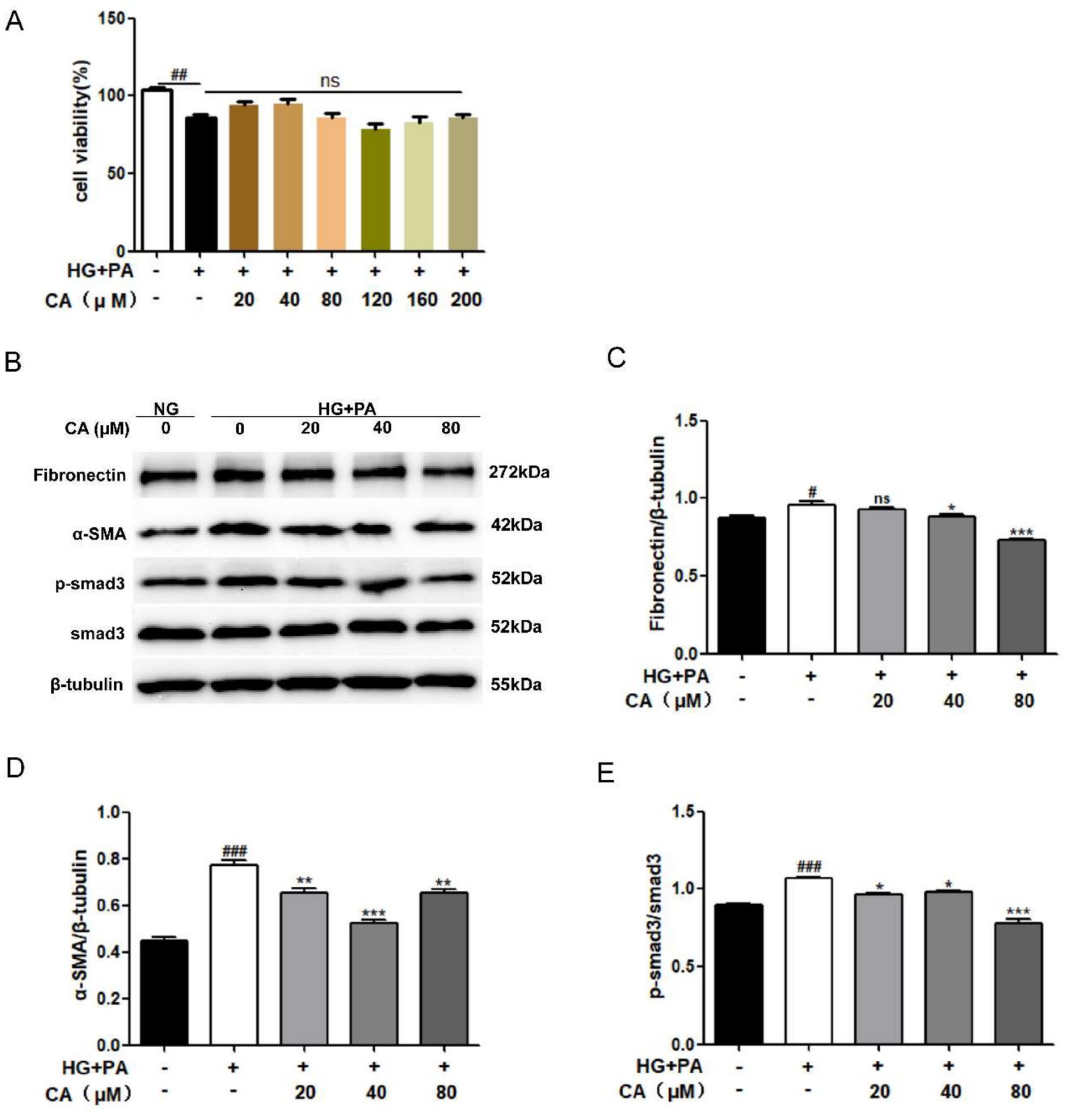
Effect of CA on fibrosis of HK2 cells induced by high glucose and high lipid. (A) HK2 cells were treated with 20, 40, 80, 120, 160, 200 μM of CA for 24 h under high glucose and high lipid conditions, and the cell viability was determined by MTT assay; (B–E) The expression of fibrosis-related proteins(Fibronectin, α-SMA, p-smad3 and smad3) in HK2 cells treated with 20, 40, 80 μM doses of CA for 24h was detected and quantified by Western blot method under conditions of high glucose and high lipid; Data are presented as means ± SEM; ^#^*p* < 0.05, ^##^*p* < 0.01, ^###^*p* < 0.001 vs. NG group; *p < 0.05, **p < 0.01, ***p < 0.001 vs. HG + PA group, *n* = 3.

Renal tubular epithelial cells HK2 were treated with different doses of chlorogenic acid (CA: 20, 40, 80, 120, 160, 200μM) for 24 h under high glucose and high lipid conditions. The results of MTT experiment showed that the cell viability of HG + PA group was decreased compared with NG group Compared with HG + PA group, cells treated with different doses of CA under high glucose and high lipid conditions did not affect cell viability (Figure 1(A)). These results suggest that CA is not toxic to cells in a hyperglycemic and hyperlipid-induced HK2 cell model. Therefore, three doses of 20, 40 and 80 μM were selected as follow-up experimental studies. We first evaluated the effect of CA on fibrosis proteins induced by high glucose and high lipid in renal tubular epithelial cells. Western blot results showed high expression of fibrosis related proteins Fibronectin, α-SMA, p-smad3/smad3, which were reversed by CA treatment. α-SMA and p-smad3/smad3 levels were significantly decreased at 20, 40, and 80 μM of CA, and 40 and 80 μM of CA markedly inhibited Fibronectin expression (Figure 1(B–E)). These results suggest that CA inhibits fibrosis of renal tubular epithelial cells induced by high glucose and high lipid levels.

### CA decreased lipid accumulation, promoted fatty acid oxidation and inhibited fatty acid synthesis in HK2 cells induced by high glucose and high lipid

**Figure 2.**
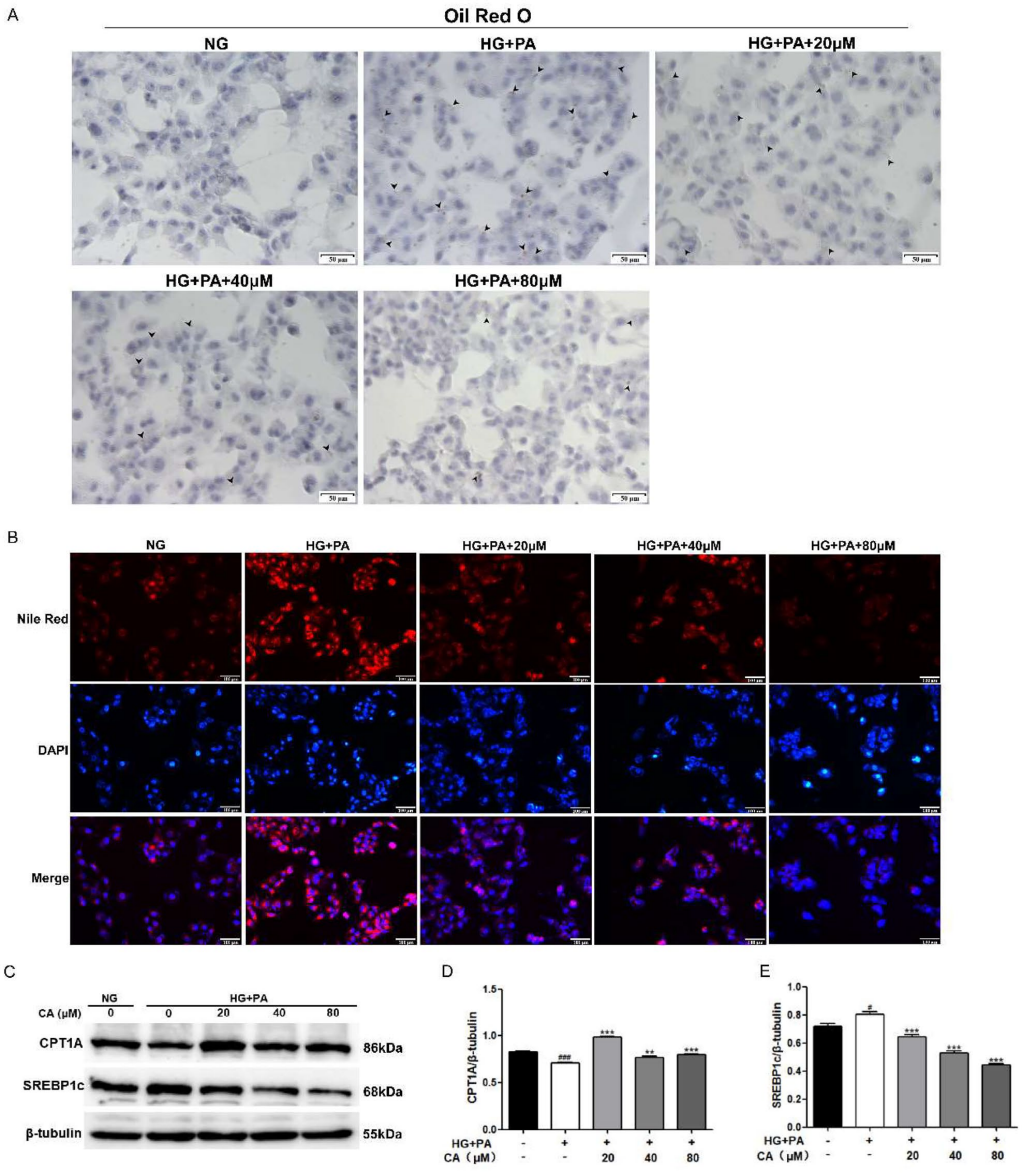
Effect of CA on lipid accumulation and fatty acid metabolism-related proteins in HK2 cells induced by high glucose and high lipid. (A) The lipid droplets (black lens) of HK2 cells treated with 20, 40, 80 μM CA for 24 h were detected by oil red O staining under high glucose and high lipid conditions; (B) The lipid droplets of HK2 cells treated with 20, 40, 80 μM CA for 24 h were detected by Nile red staining under high glucose and high lipid conditions; (C–E) the expression levels of lipid metabolization-related proteins (CPT1A and SREBP1c) in HK2 cells treated with 20, 40, 80 μM CA for 24 h were detected and quantified by Western blot method under high glucose and high lipid conditions. Data are presented as means ± SEM; ^#^*p* < 0.05, ^###^*p* < 0.001 vs. NG group; **p < 0.01, ***p < 0.001 vs. HG + PA group, *n* = 3.

At present, renal lipid accumulation has been regarded as the main pathological feature of DKD. Next, we used oil red O staining and Nile red staining methods to detect the effect of CA on the lipid accumulation in HK2 cells induced by high glucose and high lipid. The results showed that high glucose and high lipid induced intracellular lipid accumulation, and the accumulation was reversed by 20, 40, 80 μM CA treatment for 24 h (Figure 2(A and B)). At the same time, we conducted protein immunoblotting experiments on proteins related to lipid metabolism, and the results showed that high glucose and high lipid induced a decrease in the expression of fatty acid oxidation protein CTP1A and an increase in the expression of fatty acid synthesis protein SREBP1c, and these changes were reversed after 20, 40, 80 μM CA treatment (Figure 2(C–E)). These results suggest that CA reverses lipid accumulation, impaired fatty acid oxidation, and elevated fatty acid synthesis in renal tubular epithelial cells induced by high glucose and high lipid.

### CA inhibits activation of Notch1 and stat3 signaling pathways in HK2 cells induced by high glucose and high lipid

**Figure 3.**
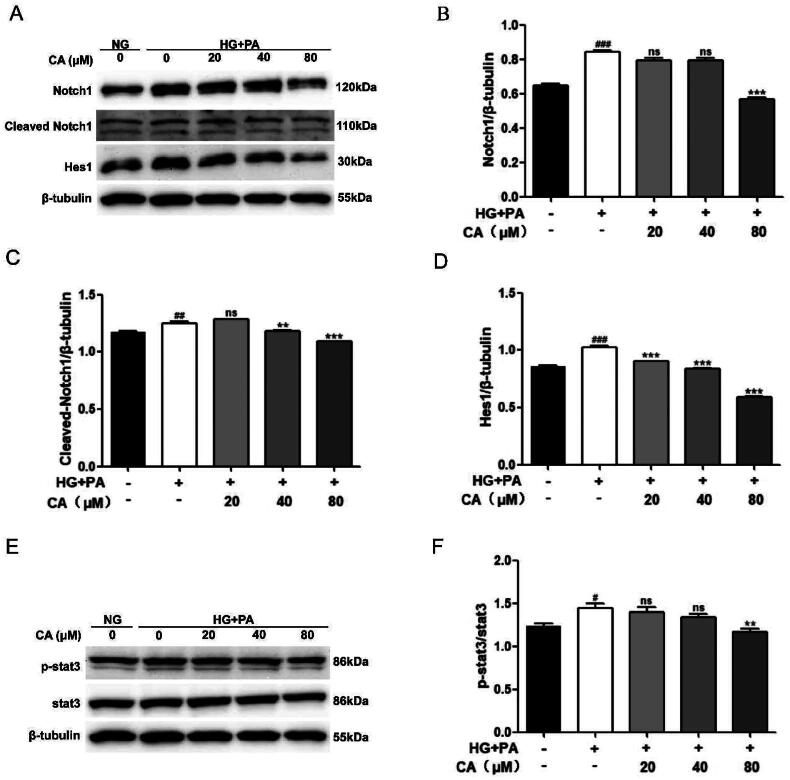
Effect of CA on Notch1 and stat3 signaling pathways in HK2 cells induced by high glucose and high lipid. (A–D) The expression levels of Notch1, Cleaved Notch1, and Hes1 in HK2 cells treated with 20, 40, and 80 μM CA for 24 h were detected and quantified by protein Western blotting; (E–F) the expression levels of stat3 and p-stat3 in HK2 cells treated with 20, 40, 80 μM CA for 24 h were detected by Western blot assay and quantified; Data are presented as means ± SEM; ^#^*p* < 0.05, ^##^*p* < 0.01,^###^*p* < 0.001 vs. NG group; **p < 0.01, ***p < 0.001 vs. HG + PA group, *n* = 3.

It has been reported that Notch1 and stat3 signaling pathways play a key role in the development of DKD, in which the continuous activation of Notch1 and stat3 signaling promotes the progression of DKD, and intervention of Notch1 and stat3 signaling is a strategy to alleviate the progression of DKD. Next, in the HK2 cell model induced by high glucose and high lipid, we detected the expression levels of Notch1 and stat3 signals-related proteins by protein western blotting. Obviously, the expression levels of Notch1, Cleaved Notch1, Hes1, p-stat3/stat3 in the HG + PA group were increased. This change is reversed after CA processing. Among them, CA reversed high glucose and high lipid induced Notch1 activation at 80 μM, reversed high glucose and high lipid induced cleaved Notch1 at 40 μM and 80 μM (Figure 3(A–D)), and reversed high glucose and high lipid induced p-stat3/stat3 activation at 80 μM (Figure 3(E–F)). Together, these results suggest that CA reverses the activation of Notch1 and stat3 signaling pathways in renal tubular epithelial cells induced by high glucose and high lipid.

### CA improves kidney injury and renal fibrosis in HFD/STZ-induced DKD mice

**Figure 4.**
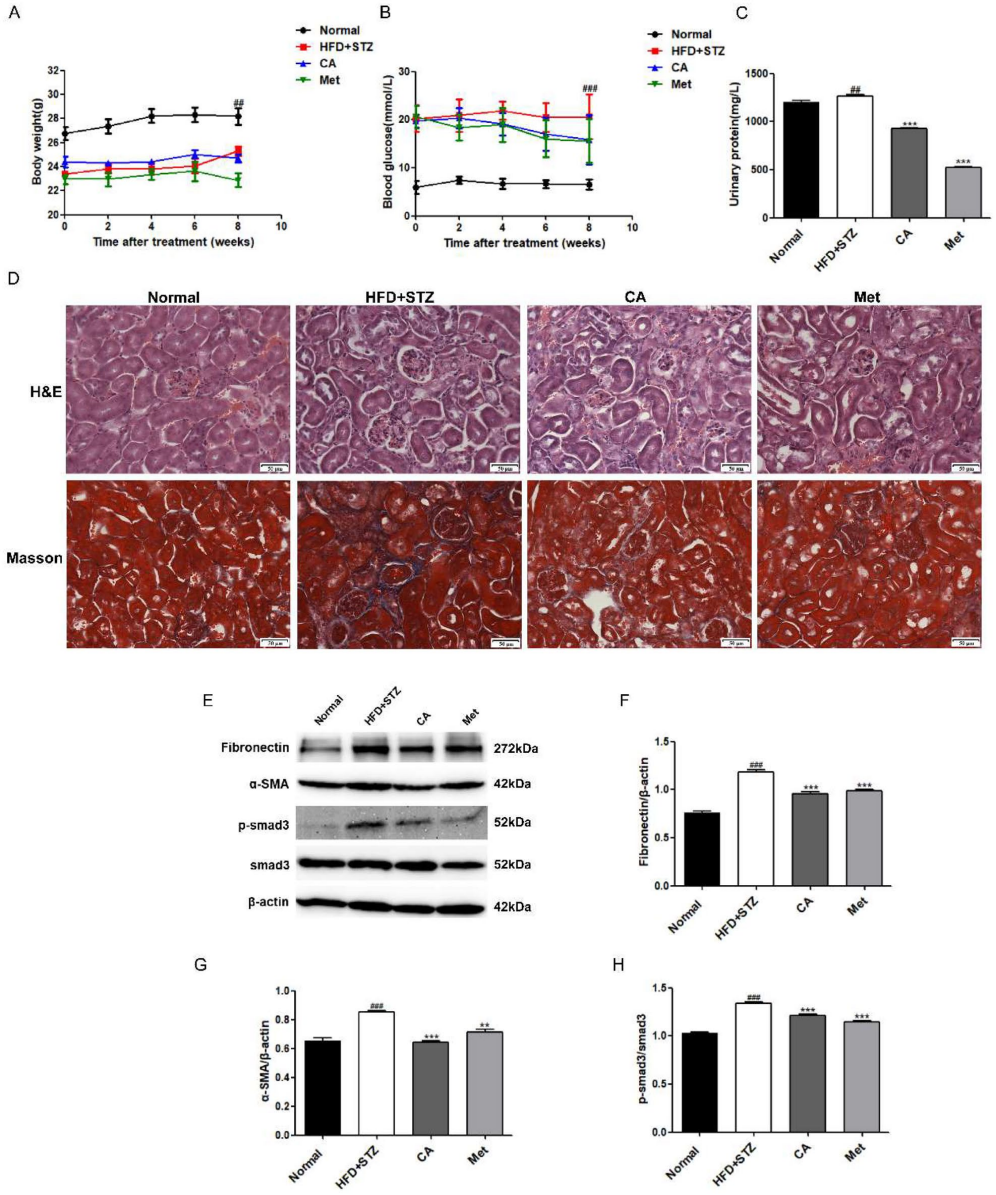
Effects of CA on renal injury and renal fibrosis in HFD/STZ-induced DKD mice. (A–C) Body weight; blood glucose; urinary protein; *n* = 6 (D) H&E and masson staining of renal tissue sections (magnification × 400); (E–H) immunoblotting and quantitative analysis of fibronectin, α-SMA, p-smad3/smad3 proteins in renal tissue; Data are presented as means ± SEM; ^##^*p* < 0.01, ^###^*p* < 0.001 vs. Normal group; **p < 0.01, ***p < 0.001 vs. HFD + STZ group, *n* = 6.

Further, in order to explore the effect of CA on DKD mice, we established C57BL/6 DKD mice by high-fat diet (HFD) and single streptozotocin intrabitoneal injection (STZ). The CA and Met canning stomach lasted for 8 weeks. The weight and blood sugar of mice were monitored every two weeks during the administration period, and urine was obtained from the metabolic cage for urine protein detection before execution. The results showed that it was obvious that the weight of mice in HFD + STZ group decreased and the blood glucose and urine protein increased. Compared with the model group, the CA and Met groups had no effect on the weight and blood glucose, but the urine protein content of CA and Met groups was significantly reduced (Figure 4(A–C)). Then, we conducted histopathological analysis experiments on the kidney tissue sections. The H&E staining results showed that glomerular basement membrane thickened and renal tubules dilated in the HFD + STZ group, indicative of kidney dysfunction. and these changes were improved to a certain extent in the CA and Met administration groups. Meanwhile, our Masson staining indicated that there was a large amount of collagen accumulation in the HFD + STZ group. However, collagen in CA and Met groups was significantly reduced (Figure 4(D)). Meanwhile, the expression levels of fibronectin, α-SMA, p-smad3/smad3, which are related to renal fibrosis, were significantly increased in HFD + STZ group, and this increase was reversed after CA and Met administration (Figure 4(E–H)). Taken together, these animal results suggest that CA improves kidney injury and renal fibrosis in HFD/STZ-induced DKD mice.

### CA improves renal lipid accumulation and lipid metabolism in HFD/STZ-induced DKD mice

**Figure 5.**
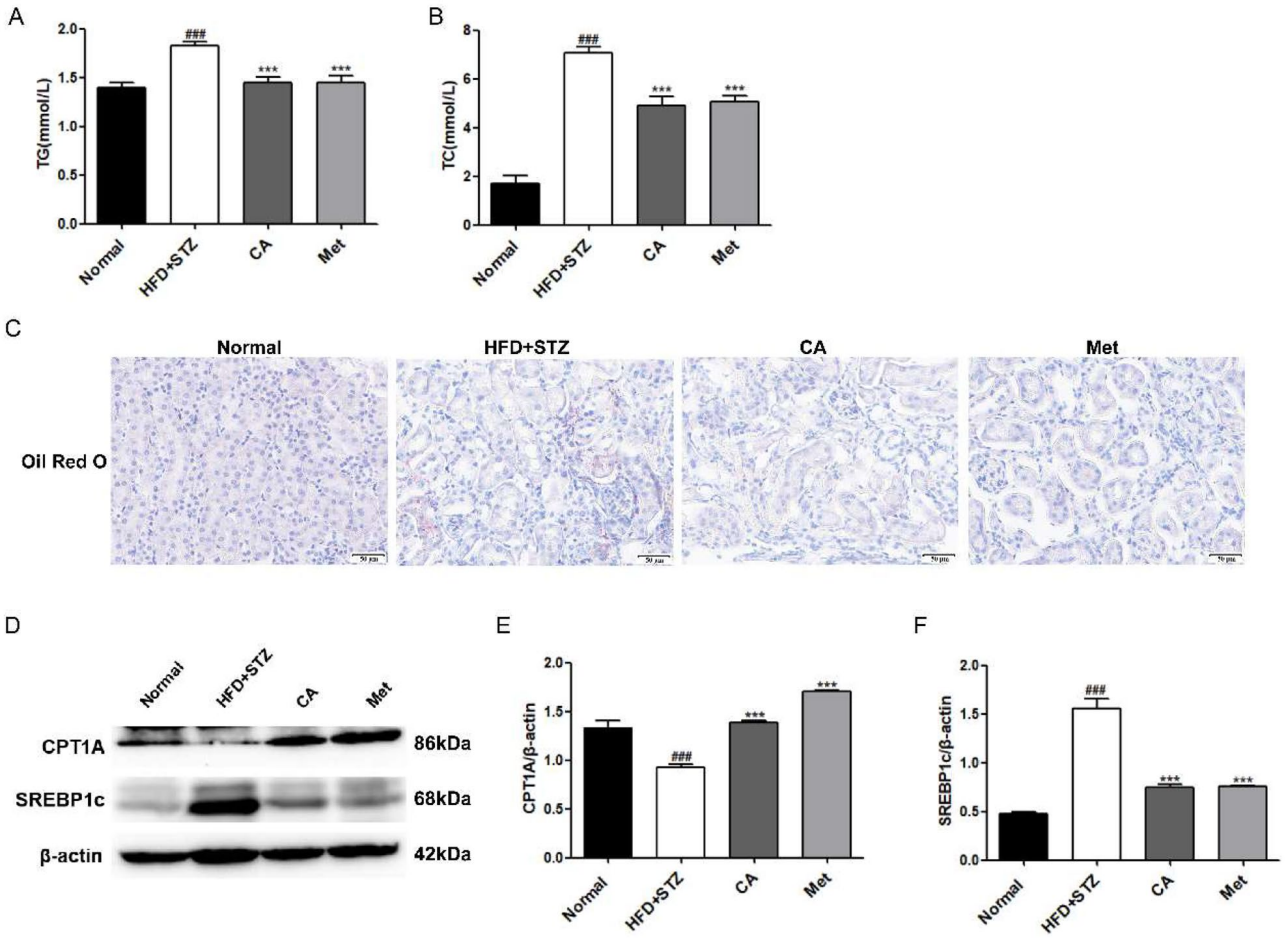
Effects of CA on serum lipids, renal lipid accumulation and lipid metabolism in HFD/STZ-induced DKD mice. (A-B) Serum TG, TC; *n* = 6 (C) Oil red O staining of frozen sections of kidney tissue; (D-F) Western blotting and quantitative analysis of CPT1A and SREBP1c proteins in renal tissue(magnification × 400). Data are presented as means ± SEM; ^###^*p* < 0.001 vs. Normal group; ***p < 0.001 vs. HFD + STZ group, *n* = 6.

Then, we detected the content of TG and TC in the serum of experimental mice, and the results showed that the content of TG and TC in the serum of mice in HFD + STZ group was significantly increased, while the content of TG and TC in the serum of mice in CA group and Met group was significantly decreased compared with that in HFD + STZ group (Figure 5(A and B)). Similarly, oil red O staining of frozen slices of mouse kidney tissue showed that HFD + STZ group had a large accumulation of lipid droplets compared with normal group mice, and this deposition was reversed by CA and Met, respectively (Figure 5(C)). Next, Western blot assay results of proteins related to lipid metabolism in mouse kidney tissues showed that the expression of fatty acid oxidizing protein CPT1A was significantly decreased and the expression of fat synthesizing protein SREBP1c was significantly increased in the kidney tissues of mice in HFD + STZ group, and this change was reversed after CA and Met administration (Figure 5(D–F)). These results demonstrate that CA improves renal lipid accumulation and lipid metabolism in HFD/STZ-induced DKD mice.

### CA inhibits Notch1 and stat3 signaling pathways in HFD/STZ-induced DKD mice

**Figure 6.**
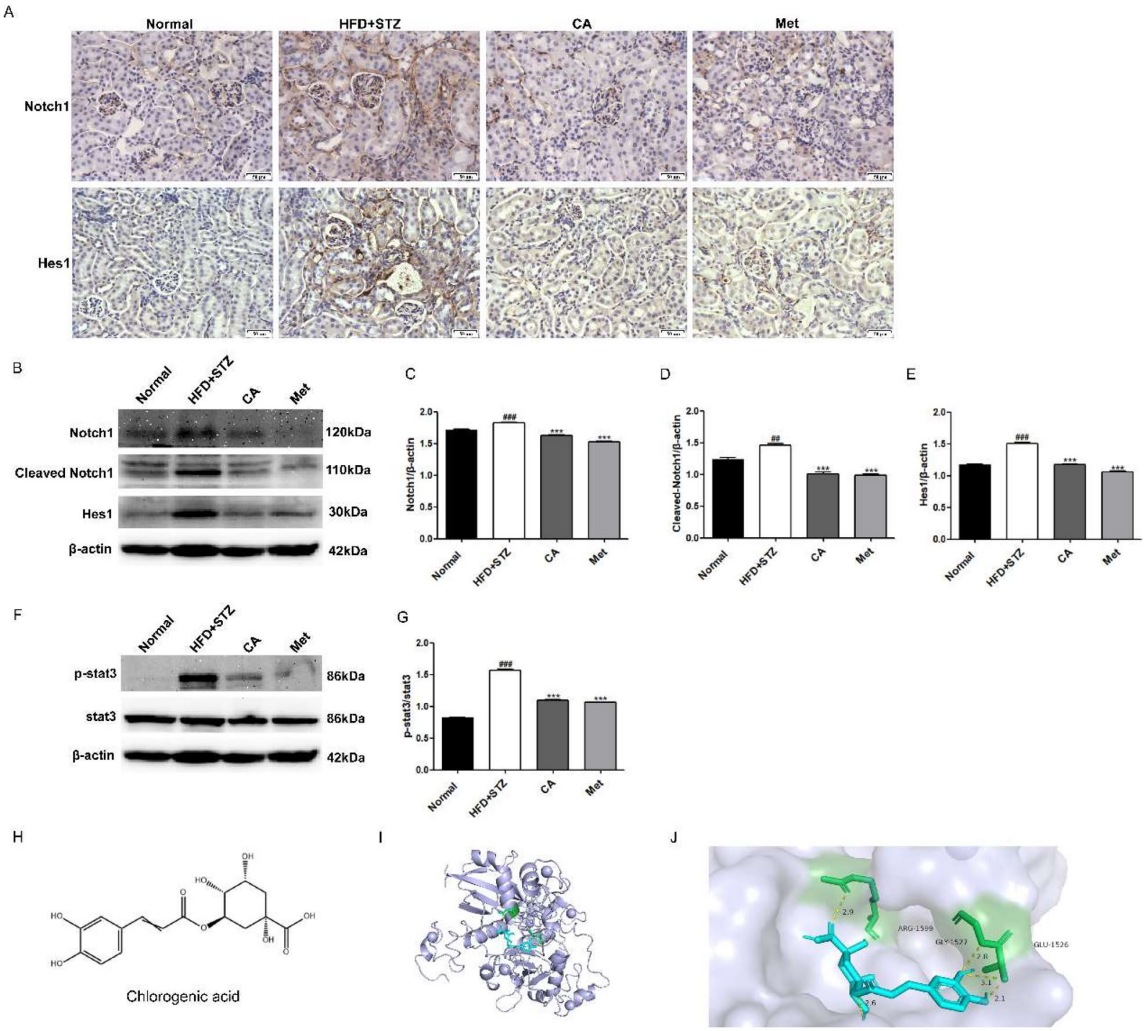
Effects of CA on Notch1 and stat3 signaling pathways in HFD/STZ-induced DKD mice and molecular docking of CA with NRR1. (A) Immunohistochemistry of Notch1 and Hes1 proteins in frozen sections of kidney tissue (brown, magnification × 400); (B-E) Western blot and quantitative analysis of Notch1, Cleaved Notch1 and Hes1 proteins in renal tissue; (F-G) Western blot and quantitative analysis of p-stat3 and stat3 protein in renal tissue; (H) The chemical structure of CA; (I) Binding models of CA (blue) in NRR1 (purple); (J) The interaction of CA with the NRR1. Data are presented as means ± SEM; ^##^*p* < 0.01, ^###^*p* < 0.001 vs. Normal group; ***p < 0.001 vs. HFD + STZ group, *n* = 6.

Finally, we measured the expression of proteins associated with Notch1 and stat3 signaling pathways in mouse kidney tissue. First, the immunohistochemical results of Notch1 and Hes1 proteins in renal tissue showed that the expressions of Notch1 and Hes1 proteins in renal tissue sections of mice in HFD + STZ group were significantly increased, while the expressions of Notch1 and Hes1 proteins in renal tissue sections of CA and Met groups were decreased (Figure 6(A)). Second, western blot results of Notch1, Cleaved Notch1, and Hes1 proteins showed significant increase in their expression in HFD + STZ mice, and this high expression was reversed by the compounds CA and Met, respectively (Figure 6(B–E)). At the same time, p-stat3/stat3 protein levels were also significantly increased in the kidney tissues of HFD + STZ group, while compounds CA and Met inhibited p-stat3/stat3 expression levels, respectively (Figure 6(F and G)). Finally, in order to further explore the regulatory role of CA on Notch1, we simulated the possible interaction between CA and the extracellular domain NRR1 of Notch1 protein by molecular docking method. The molecular docking results showed that the interaction between CA and NRR1 was mainly hydrogen bond interaction. The binding energy of CA and NRR1 is −6.6 kcal/mol, and it is preliminarily predicted that CA may combine with NRR1 to regulate Notch1 signal (Figure 6(H–J)). In conclusion, CA inhibits the activation of Notch1 and stat3 signaling pathways in kidney tissue of HFD/STZ-induced DKD mice.

## Discussion

With the change of people’s eating habits and lifestyle, more and more people are accompanied by various metabolic diseases such as diabetes. The long-term high blood sugar, high fat and high blood pressure environment of diabetic people for many years will lead to various complications such as DKD.CA is a natural, low toxicity, a wide range of small molecular phenolic compounds, which are mainly derived from fruits, vegetables and Chinese herbs [11], such as coffee, mulberry and other foods with high content. Previous studies have shown that CA has been used to prevent and treat diabetes and various complications [32]. Among them, in experimental diabetic rats, CA decreased renal MDA levels, increased SOD and GSH-Px activities, and decreased the expression of factors related to renal oxidative stress and inflammatory response. In this study, pathological examination found that CA reduced glomerular hypertrophy and mesangial matrix expansion [18], and another study had similar results [33]. It is suggested that CA can prevent and treat DKD by alleviating renal oxidative stress and inflammatory response, but the specific molecular mechanism is still unclear. In addition to inflammation, oxidative stress, renal tubulointerstitial fibrosis, and the role of renal lipid accumulation in DKD has been extensively studied [34, 35], Studies have found that CA has a role in regulating lipid metabolism, and in Wistar rats exposed to a high-sugar, high-fat diet, CA improved liver lipid metabolism [36], CA significantly reduced lipid-related markers in the liver and kidneys [37]. However, no studies have been reported on the role of CA in lipid accumulation and lipid metabolism in DKD.

In this study, we first exposed renal tubular epithelial cells HK2 to conditions of high glucose (D-glucose) and high lipid (PA) to assess the effect of diabetes-induced high glucose and high lipid environment on fibrosis and lipid deposition of HK2 cells and the role of CA in this. Our results show that significantly increased expression levels of the fibrotic protein fibronection, extracellular matrix protein α-SMA, and the classical protein p-smad3/smad3 representing fibrogenesis were observed in HK2 cells induced by high glucose and lipid, however, this change was significantly reversed after treatment of the cells with three different doses of CA (Figure 1(B–E)), except for α-SMA, CA inhibited their expression in a dose-dependent manner, suggesting that CA inhibited hyperglycemic and hyperlipid-induced tubular epithelial cell fibrosis. A large number of literatures have reported [38–41] that TGF-β1/Smad signaling pathway is the classic pathway of renal fibrosis. This study not only proves the improvement of fibrosis markers by CA, but also proves the inhibition of TGF-β1/Smad signaling pathway by CA. Secondly, cell oil red O staining and Nile red staining showed that lipid droplets significantly increased in HK2 cells exposed to high glucose and high lipid conditions, and CA reduced the amount of lipid droplets accumulation in a dose-dependent manner (20 μM,40μM, 80 μM) (Figure 2(A and B)). Meanwhile, fatty acid synthesis and oxidation, as two key steps of lipid metabolism, are related to lipid deposition. Impaired fatty acid oxidation and increased synthesis promote lipid accumulation. Cholesterol regulatory element binding protein (SREBP) is the main regulator of fatty acid and cholesterol synthesis in ectopic lipid accumulation [42], Carnitine palmitoyl transferase 1 A(CPT1A) is the main regulatory enzyme of fatty acid oxidation [43]. Based on this, we performed western blotting on proteins related to these two processes, and the results showed that the expression of CPT1A protein related to fatty acid oxidation and SREBP1c protein related to fatty acid synthesis were decreased in HK2 cells induced by high glucose and high lipid. CA significantly reversed the decrease of CPT1A and the increase of SREBP1c (Figure 2(C–E)). Studies have reported that the decreased expression level of fatty acid oxidation regulatory enzyme in renal tubular epithelial cells of diabetic patients contributes to intracellular lipid accumulation and the development of renal fibrosis[43], and the overexpression of SREBP1 in the renal tissues of diabetic mice leads to renal lipid accumulation, renal tubular interstitial fibrosis and glomerulosclerosis[42]. Taken together, these results suggest that CA may reduce lipid accumulation by regulating abnormal expression of CPT1A and SREBP1c, thereby ameliorating hyperglycemic and hyperlipid-induced renal tubular epithelial fibrosis.

Multiple signaling pathways are involved in the development of DKD. Among them, the continuous activation of Notch and JAK/STAT signaling promotes DKD and renal fibrosis [44], Notch signaling is a transmembrane signal. There are mainly 4 membrane proteins (Notch1-4). After ligands bind to receptors and are cleaved by multiple proteases, they are ectopic to bind to transcription factor Hes1 in the nucleus and then regulate the process of disease development. Currently, Notch1-Hes1 axis has been widely studied in DKD. It was also found that the binding of Notch1 signal target gene Hes1 to stat3 mediated the interaction between Notch1 signal and stat3 signal, and indicated that inhibition of Hes1 could inhibit the phosphorylation of stat3 [28]. Inhibition of Notch signaling can regulate lipid metabolism and improve nonalcoholic fatty liver disease. The pro-fibrotic effect of Notch signaling is related to impaired lipid metabolism of renal tubular epithelial cells [24], and regulation of stat3 signaling can promote the oxidation of fatty acids and improve lipid accumulation in liver [27]. In this study, we hypothesized that CA could inhibit Notch1 and stat3 signaling, and that CA might regulate Notch1 and stat3 signaling to inhibit renal lipid accumulation and renal fibrosis induced by high glucose and high lipid in HK2 cells. To verify our hypothesis, we performed protein immunoblot studies on the related proteins of the two pathways. Notably, CA inhibited the expression levels of high-glucose and high-lipid induced Notch1, Cleaved Notch1, Hes1, and p-stat3/stat3 in HK2 cells (Figure 3). It is suggested that CA may reduce renal lipid accumulation by inhibiting Notch1 and stat3 signaling induced by high glucose and high lipid, thereby improving renal fibrosis.

In order to further verify whether CA has a consistent effect *in vivo*, we established DKD in type 2 diabetic mice induced by HFD/STZ. It is worth noting that although CA has no hypoglycemic effect in mice, it can significantly improve urinary protein, renal tissue morphology (glomerular basement membrane thickening, interstitial expansion) and collagen accumulation. The levels of fibrosse-related proteins were also significantly improved, suggesting that CA can improve kidney injury and kidney fibrosis in DKD mice, which is consistent with our *in vitro* experiment and the research results of Liping Bao et al. [18]. In addition, Met, as a positive drug, did not show a significant hypoglycemic effect in this study, which may be due to the low dose we used (50 mg/kg), and generally 100-200mg/kg dose is required. However, Met has a significant improvement effect on renal injury and fibrosis in DKD (Figure 4). In addition, the increase of serum indexes including TG and TC levels in mice was reduced after CA treatment, and the accumulation of kidney lipid droplets was significantly reduced by oil red O staining in kidney tissue (Figure 5(A–C)). which was consistent with the regulation of liver lipid metabolism by CA studied by Xiang et al. [14]. Protein western blotting proved that CA could reverse the low expression of CPT1A and high expression of SREBP1c in renal tissue of DKD mice, which was consistent with the results *in vitro*, and further verified our conclusion that CA could improve renal fibrosis by reducing renal lipid accumulation in DKD mice (Figure 5(D–F)). Then, we measured the expression levels of Notch1 and STAT3-related proteins in kidney tissue, and CA significantly inhibited the expression of Notch1 and stat3 signal-related proteins in kidney tissue of DKD mice (Figure 6(A–G)). Our previous *in vitro* and *in vivo* studies respectively demonstrated the inhibitory effect of CA on Notch1 signaling pathway. In order to further explore and predict the effect of CA on Notch1 protein extracellular domain NRR1, we conducted molecular docking experiments of CA and Notch1 protein extracellular domain NRR1. Obviously, the binding capacity of CA and NRR1 is −6.6 kcal/mol. It was preliminarily indicated that CA may act on the extracellular domain NRR1 of Notch1, and the specific site of action needs to be further studied (Figure 6(H–J)).

**Figure 7.**
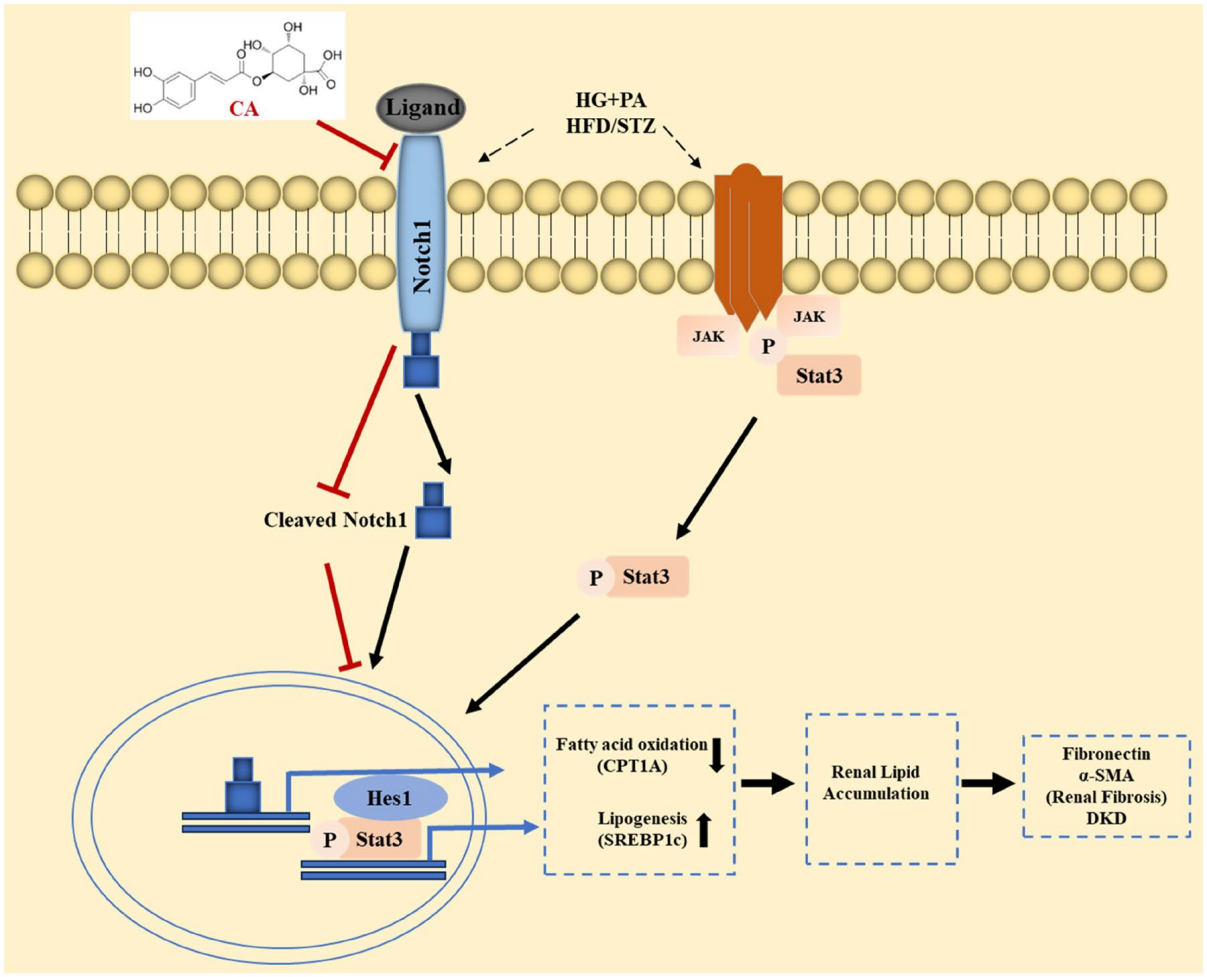
Chlorogenic acid alleviates renal fibrosis by reducing lipid accumulation in DKD through suppressing the Notch1 and Stat3 signaling pathway. CA inhibition of Notch1 and stat3 signaling weakens SREBP1C-mediated lipogenesis and increases fatty acid oxidation by increasing CPT1A expression. Decreased fatty acid synthesis and increased fatty acid oxidation reduce lipid accumulation in renal tubular epithelial cells, thereby improving renal fibrosis in DKD.

In conclusion, in the high-glucose and high-lipid induced HK2 cell model and the kidney tissue of HFD/STZ-induced DKD mice, CA reduces renal lipid accumulation by inhibiting the activation of Notch1 and stat3 signaling pathways, thereby improving DKD renal fibrosis (Figure 7). The discovery of this mechanism provides a theoretical basis for the application of natural plants containing CA in chronic diseases such as DKD, and also lays a research foundation for the use of natural compounds CA in the prevention of human DKD.

## Data Availability

The raw data supporting the conclusion of this article will be made available by the authors, without undue reservation.

## Abbreviations

(DKD): diabetic kidney disease
(CA): chlorogenic acid
(α-SMA): α-smooth muscle actin
(NG): normal glucose
(HG): high glucose
(PA): palmitic acid
(Met): metformin
(HFD): high fat diet
(STZ): streptozotocin
(CPT1A): carnitine palmitoyl transferase 1A
(SREBP1c): cholesterol regulatory element binding protein 1c.
